# Soft-tissue tension during total hip arthroplasty measured in four patients and predicted using a musculoskeletal model

**DOI:** 10.1186/s40634-023-00689-7

**Published:** 2023-12-05

**Authors:** Masaru Higa, Hiromasa Tanino, Hiroshi Ito, Scott A. Banks

**Affiliations:** 1 Department of Mechanical Engineering, University of Hyogo, Shosha2167, HimejiHyogo, 671-2280 Japan; 2 Department of Orthopaedic Surgery and Arthroplasty, Asahikawa Medical University, Asahikawa, Hokkaido Japan; 3 Department of Mechanical & Aerospace Engineering, University of Florida, Gainesville, FL USA

**Keywords:** Dislocation, Total hip arthroplasty, Soft-tissue tension, Sensor, Joint force

## Abstract

**Purpose:**

Soft-tissue tension around the hip joint is related to the incidence of dislocation after total hip arthroplasty (THA), but it remains difficult to quantify the soft-tissue tension during surgery. In this study, a three-dimensional force sensor-instrumented modular femoral head was developed and used to quantify soft-tissue tension during THA. The forces at the hip joint were also calculated using a three-dimensional musculoskeletal computer model to validate the measured forces.

**Methods:**

Soft-tissue tension was investigated by measuring the hip joint forces and directions during intraoperative trialing in four patients through passive range of motion (ROM) from 0° extension to 90° flexion. A musculoskeletal model with THA, which was scaled to one of four patients, was developed. The hip joint forces were calculated under the same motion.

**Results:**

Through the passive ROM, the magnitude of soft-tissue tension was greatest when the hip was extended, decreased with flexion to 34°, and progressively increased to flexion at 90°. The mediolateral force component was relatively constant, but the supero-inferior and anterior–posterior force components changed significantly. Within-individual variations were small during three repeated cycles of measurement, but magnitudes varied significantly among patients. Similar force patterns and magnitudes were calculated by the musculoskeletal model.

**Conclusions:**

This study demonstrates that it is possible to quantify soft-tissue tension and direction during THA with an instrumented head. There was general agreement between the calculated and measured forces in both pattern and magnitude. Including additional subject-specific details would further enhance agreement between the model and measured hip forces.

## Introduction

Dislocation occurs in 2% to 11% of primary total hip arthroplasties (THAs) and more than 60% of the patients who experienced dislocation once suffered from recurrent dislocation [3, 14, 16, 33]. Revision surgery is needed to correct the instability in 22% to 51% of such cases [11, 16, 33]. The risk factors that affect dislocation include soft-tissue laxity, surgical approach, component malposition, patient factors, and component design [4, 11, 18, 24, 26, 27]. In terms of revision surgery, based on the latest THA databases from England, Wales, Northern Ireland, the Isle of Man and the States of Guernsey, the most common indications for revision were aseptic loosening (35%), periprosthetic fracture (22%), infection (21%) and dislocation/subluxation (18%). In the short term especially (less than 1 year after primary THA), the most common indication was dislocation [23]. In the United States, on the other hand, dislocation is the most common cause of revision surgery after THA [7]. There are wide variations of these numbers due to differences in study design, but it is clear that dislocation after THA remains an important complication that surgeons may be able to diminish.

Although many factors affect the incidence of dislocation, in vivo or in vitro studies suggest that soft-tissue tension could be a major cause of subluxation or dislocation after THA [1, 22, 25, 29]. Thus, achieving proper soft-tissue balance has been an explicit goal of THA surgery. Intraoperative soft-tissue tension can be quantified by joint contact force measurements using an instrumented prosthesis [13, 28]. However, few attempts have been made to measure hip joint forces intraoperatively during THA [20], and the optimal hip joint force pattern remains unknown. In other words, relationships between intraoperative hip joint forces and postoperative hip function remain unknown, which makes it difficult to determine desired ranges for intraoperative hip joint forces resulting from soft-tissue tension by changing component size and placement. Based on the results of in vitro studies, however, researchers have hypothesized that forces acting near the rim of the polyethylene liner will cause dislocation during flexion and/or adduction [1, 12].

An additional method of validating the measured hip joint forces is to compare the measured forces with calculated hip joint forces using a musculoskeletal computer model. This comparison will make it possible to determine whether in vivo measurements are within the range of those found in the calculated results [19]. OpenSim is a widely used musculoskeletal modeling package that allows users to manipulate model parameters to analyze the biomechanical consequences of orthopaedic procedures [5].

The objectives of this study were (1) to demonstrate that a new sensor could be used intraoperatively, (2) to quantify three-dimensional hip joint forces during intraoperative trialing maneuvers, and (3) to validate the measured hip joint forces using a musculoskeletal computer model.

## Materials and methods

### Sensor-instrumented prosthesis

**Fig. 1:**
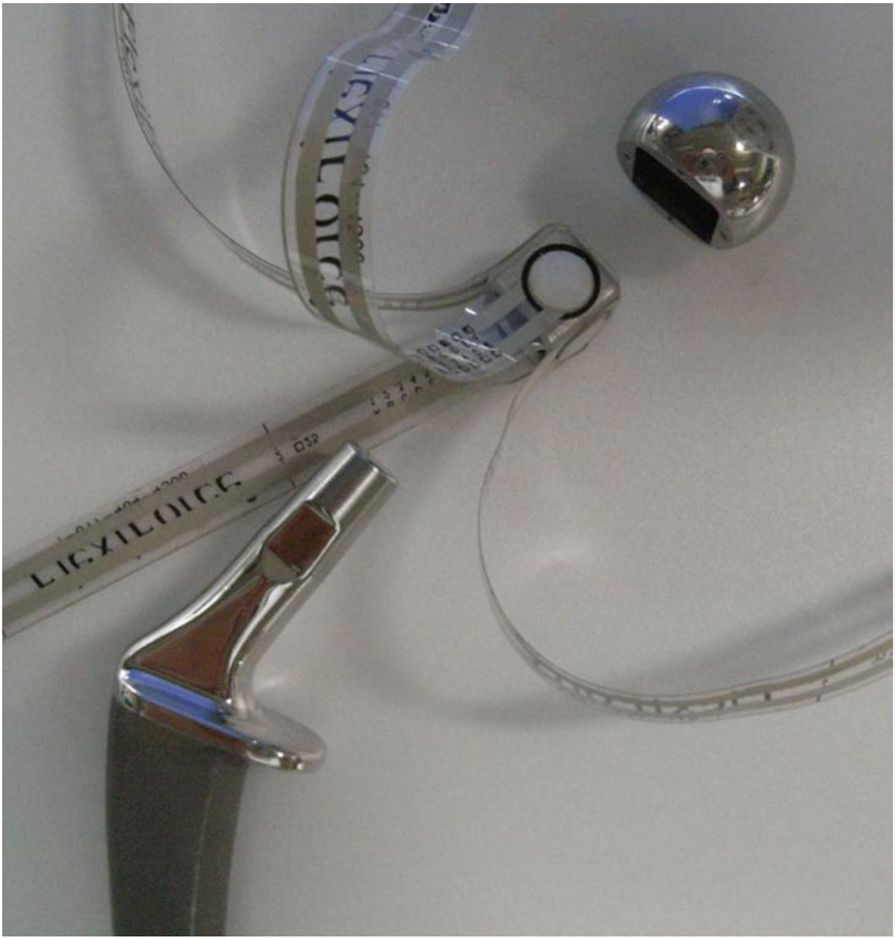
A photograph of an instrumented prosthesis, which is reassembled, and a stem. Four pressure sensors are placed on the faces of a cube-shaped part

**Fig. 2:**
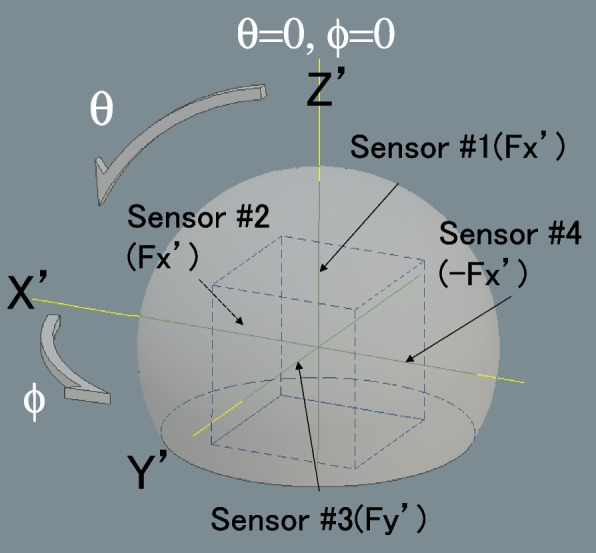
This schematic illustration represents the positions of each sensor and the definitions of the femoral head coordinate axes

**Fig. 3:**
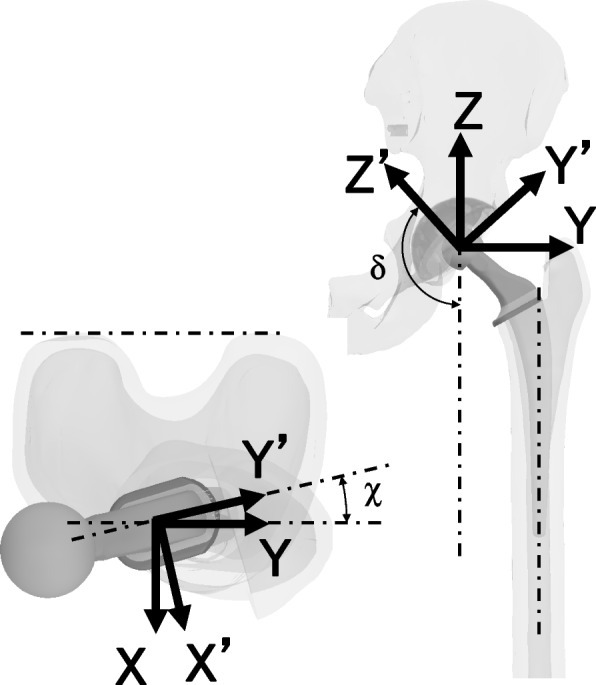
The femoral head coordinate system is represented in gray (X, Y, Z), while the pelvic coordinate system is represented in black (X’, Y’, Z’). The X axis is not shown, which faces anteriorly

The sensor-instrumented modular femoral head was composed of two metal parts (hemispherical metal part and cube-shaped metal part) made of stainless steel with four pressure sensors (FlexiForce A201-100, Tekscan, Inc., Boston, MA, USA) (Fig. 1). The diameter of the hemispherical metal part was 26 mm, and the cube-shaped metal part had 14-mm-long edges with a tapered hole at the bottom for stem insertion. Sensors were placed on the faces of the cube-shaped metal part, so that three mutually perpendicular force components could be measured, e.g., Sensor #1 measures the Fz’ (θ = 0, θ = 0) force component only (Fig. 2). This instrumented modular femoral head was attached to the neck of the femoral stem instead of the real femoral head or trial head during surgery. When the head was attached to the stem in vivo, the Y’ axis was facing the lateral-superior direction (Figs. 2 and 3). The stem neck inclination angle (δ), flexion angle of the hip (γ), stem varus angle relative to the femur (ɛ), and anteversion angle (χ) were quantified. The angle δ was constant at 130° for all measurements, γ was measured intraoperatively by a camera during trialing maneuvers, ɛ was measured on postoperative anteroposterior radiographs, and χ was measured on postoperative computed tomography (CT). Three-dimensional force-sensing trial head coordinate system O’ (X’, Y’, Z’) were transferred to the pelvic coordinate system O (X, Y, Z). which was fixed to the body (Fig. 3). The three-dimensional forces ***F’*** (Fx’, Fy’, Fz’) were measured in the implant-base coordinate system O’ (X’, Y’, Z’). For transforming the force ***F’*** to the ***F*** in the pelvic coordinate system O (X, Y, Z), transformations have to be performed, using the transformation matrices. Rotations around the axes x y z are performed by these matrices.

1 $$\begin{array}{c}{T}_{x}=\left(\begin{array}{ccc}1& 0& 0\\ 0& \mathrm{cos}{\alpha }_{x}& -\mathrm{sin}{\alpha }_{x}\\ 0& \mathrm{sin}{\alpha }_{x}& \mathrm{cos}{\alpha }_{x}\end{array}\right) {T}_{y}=\left(\begin{array}{ccc}\mathrm{cos}{\alpha }_{y}& 0& \mathrm{sin}{\alpha }_{y}\\ 0& 1& 0\\ -\mathrm{sin}{\alpha }_{y}& 0& \mathrm{cos}{\alpha }_{y}\end{array}\right) { T}_{z}=\left(\begin{array}{ccc}\mathrm{cos}{\alpha }_{z}& -\mathrm{sin}{\alpha }_{z}& 0\\ \mathrm{sin}{\alpha }_{z}& \mathrm{cos}{\alpha }_{z}& 0\\ 0& 0& 1\end{array}\right)\end{array}$$

The stem neck inclination angle (δ) was stated relative to the O’, while γ, ɛ and χ were stated relative to the O. Thus, for transforming the force vector, the rotation matrices were applied:

2 $$\begin{array}{c}F={T}_{y}\left(\gamma \right){T}_{z}\left(\chi \right){T}_{x}\left(\varepsilon \right){T}_{{x}{\prime}}\left(\delta \right){{\varvec{F}}}^{\boldsymbol{^{\prime}}}\end{array}$$

The sterilized sensors and other non-sterilized devices, including a data logger, analog-to-digital converter, and a personal computer were connected prior to surgery. Output signals from each sensor were recorded at a frequency of 100 Hz by the data logger.

### Intraoperative measurements

Four female patients gave written, informed consent for intraoperative hip force measurement using the sensor-instrumented modular femoral head. This study was approved by the Institutional Review Board. The first patient underwent THA for rheumatoid arthritis (RA), and the remaining patients underwent THA for osteoarthritis (OA) secondary to developmental dysplasia of the hip. According to the classification system of Hartofilakidis et al. [9], all three OA hips were Type 1 (dysplasia). The first and third patients were small and light compared with the patients in our previous report of 71 patients whose mean height and weight were 153 cm (136 to 170 cm) and 58 kg (41 to 95 kg) [14]. Preoperative average flexion was 80° (40° to 100°), and extension was 0° in all cases. Preoperatively, the operated limb in all patients was shorter than the contralateral limb on radiological evaluation. Leg-lengthening averaged 16 mm (11 to 22 mm), and the mean postoperative difference in leg-length discrepancy between the operated and contralateral limb was 3 mm (1 to 5 mm) (Table 1). The degree of leg lengthening was assessed radiographically. A horizontal line was drawn through points at the most inferior aspect of the acetabular teardrop of each hemipelvis, and the center of the lesser trochanter was taken as the corresponding reference point on the femora. The distances from the femoral reference point to a perpendicular intersection with the pelvic reference line in the preoperative and postoperative radiographs were measured. Pre-existing leg-length discrepancy was accounted for during preoperative planning and was corrected at the time of THA. The degree of lengthening of an operated limb and the postoperative leg-length discrepancy between the operated and contralateral limbs were measured. Two patients (first and second patients) had undergone bilateral hip arthroplasty, so the postoperative leg-length discrepancies in these two patients were comparisons of the operated hip to the contralateral previously operated hip.

**Table 1:** Demographic characteristics of the four patients

Diagnosis		Age (yr)	Height (cm)	Weight (kg)	Preoperative ROM (flex/ext)	Leg-lengthening (mm)	Postoperative leg-length discrepancy (mm)
Patient 1	RA	77	136	40	40/0	+ 19	+ 1
Patient 2	OA	65	152	76	100/0	+ 11	-5
Patient 3	OA	80	139	36	100/0	+ 11	+ 4
Patient 4	OA	58	151	58	80/0	+ 22	-3
Precious report (Ito et.,2003) [14]		62(26–80)	153(136–170)	58(41–95)			

**Fig. 4:**
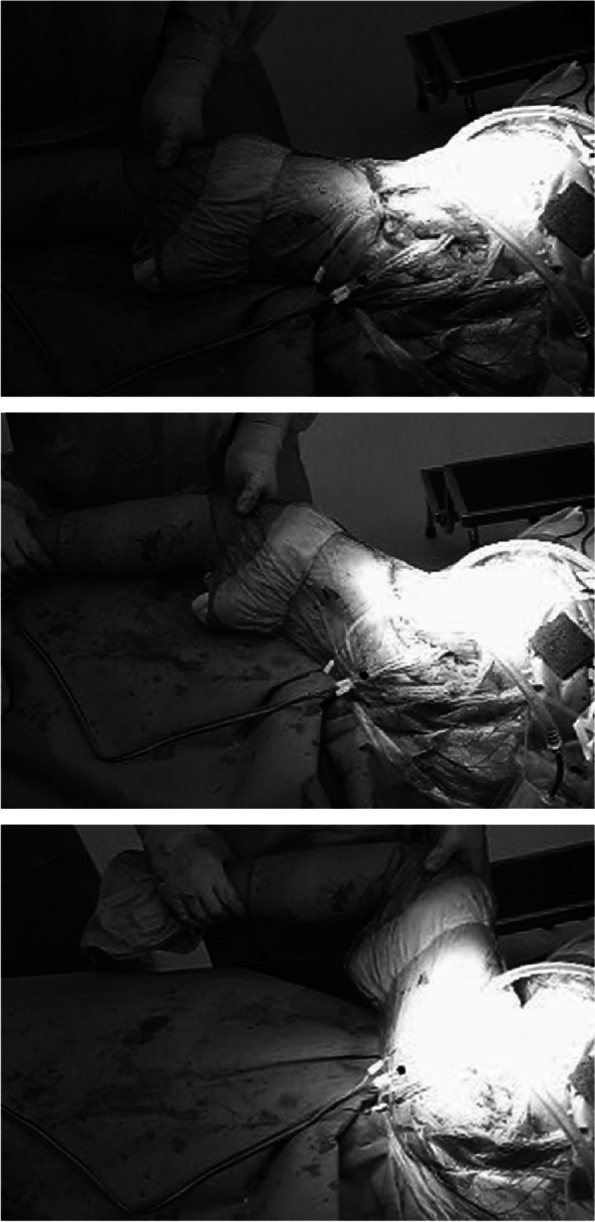
Photographs show intraoperative measurements of hip forces. The electric wires from the sensors are connected to the control parts (not shown). Three photographs represent the movements considered in this study from extension (top) to flexion (bottom)

All procedures were performed by the same senior surgeon. Hybrid prostheses (cementless acetabular component and a cemented femoral component) of one design (4-U; Nakashima Medical, Okayama, Japan [15, 30]), 26-mm femoral head, and a posterolateral approach without trochanteric osteotomy or posterior capsular repair were used in all cases. Detailed surgical techniques for OA secondary to developmental dysplasia of the hip have been reported previously [14]. The neck length and offset were determined based on preoperative templating, and then minor adjustments were made intraoperatively when necessary to optimize abductor tension and joint stability. After all components except the femoral head were placed, the sensor-instrumented modular femoral head was attached to the neck of the femoral stem. With the sensor-instrumented modular femoral head in place, the hip and the knee were simultaneously taken through passive ROM from 0° extension to 90° flexion with 0° abduction/adduction three times (Fig. 4). The movements were captured by a standard video camera. The magnitudes and directions of measured forces were recorded throughout the range. After measurements, the sensor-instrumented modular femoral head was removed from the neck of the femoral stem, and the final 26-mm metal femoral head was attached.

### Statistics

Intrasubject variability was determined by calculating the coefficient of variation (COV%) from three cycles in each patient (COV% = 100*standard deviation/mean). Mean intrasubject COV% was calculated from the COV% of each patient. The intersubject variability was determined by calculating COV% from the total number of patients.

### Musculoskeletal model simulation

**Fig. 5:**
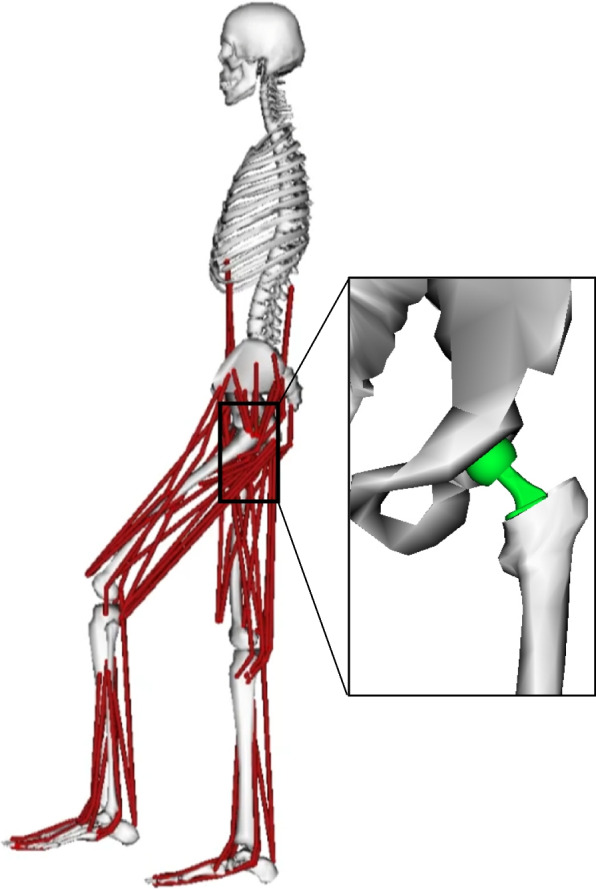
A musculoskeletal model with THA is shown. Only the muscles related to the lower limb are shown. Size and weight were scaled to patient 4

A three-dimensional musculoskeletal model of the lower limb with all relevant hip musculature was used to predict the hip joint forces for patient 4. This model was constructed using musculoskeletal software (OpenSim) with a generic model (gait2372) focusing on detailed geometries and parameters of muscles spanning the hip [5, 6]. The torso and lower body were represented as 10 segments with 23 degrees of freedom (DOFs). The hip was represented as a gimbal joint (3 DOFs), and the knee was represented as a pin joint (1 DOF). Twenty muscles spanned each hip in the model. Bone geometries were scaled to the anatomic dimensions of the patient based on postoperative anteroposterior radiographic views of bilateral hips. Only patient 4 was modeled to predict the hip joint forces. After scaling the bone geometries, the joint position and the femur position relative to the pelvis were adjusted, assuming that the THA components for the subject were implanted in the femur (Fig. 5). The pelvis in this model was fixed to the ground, whereas the femur and tibia were allowed to move via the hip and knee joints. The hip joint forces were defined by the force exerted by the femoral head to the pelvis relative to the pelvis coordinate system (X, Y, Z in Fig. 3) so that the forces could be compared to the forces measured intraoperatively. To simulate passive muscle function following THA (posterolateral approach), some external rotator muscles were omitted from the model [8]. This patient-specific hip model was used to calculate hip joint forces caused by passive muscle forces during passive movements. Muscle excitations were set to zero to simulate a condition under anesthesia. External hip joint moments were applied to the femur and the tibia to satisfy static equilibrium for each recorded position during passive ROM trials. For the hip joint, a joint force vector was set equal to and opposite to the sum of the vectors of the forces of muscles crossing the hip, including some two-joint muscles and the gravitational forces acting on the lower limb [32]. Since the movements used in this study were slow, inertial forces were excluded from the analysis.

## Results

### Intraoperative measurements

**Fig. 6:**
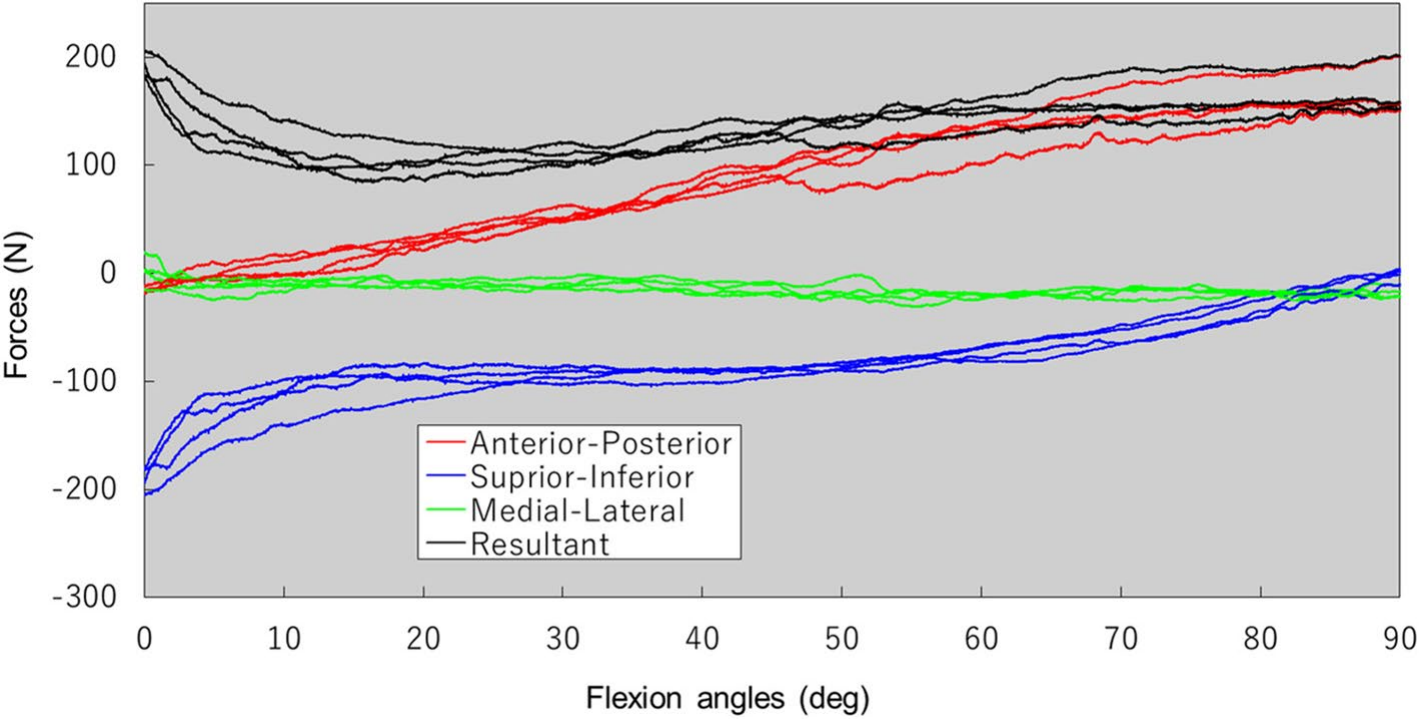
Dynamic hip joint forces are measured from extension to flexion. Three directions agree with the pelvic coordinate system (Fig. 3). Positive values correspond to forces directed posteriorly, inferiorly, and medially

Dynamic soft-tissue tension and direction were successfully measured during surgery using the sensor-instrumented modular femoral head in all four patients. Table 2 shows the measured forces, and Fig. 6 demonstrates the forces in patient 4. Positive values mean the forces are directed posteriorly, inferiorly, and medially. In all four patients, the hip force was greatest, averaging 419 N (183 to 804 N) at the most extended posture. The force decreased with increasing flexion angle and reached a minimum, averaging 120 N (85 to 143 N), at a mean flexion of 34° (11 to 50°). The force again increased with further flexion to 90°, averaging 205 N (153 to 284 N) at the most flexed posture. From extension to flexion, the medial–lateral force component changed little, but superior-inferior and anterior–posterior force components changed in all four patients. With hip flexion, the superior component decreased, while the posterior component increased. The within-individual force direction and the pattern were mostly small during three repeated cycles of measurement in each patient (Fig. 6). The COV of the maximum resultant hip force in the extended hip was 7% (2 to 14%), whereas the minimum force was 7% (2 to 10%), and the force at flexion of 90° was 5% (2 to 7%) during three repeated cycles of measurement in each patient. However, the between-individual COVs of the forces were large, especially for the maximum resultant hip force in the extended hip. The COV of the maximum resultant hip force in the extended hip was 92%, the minimum force was 29%, and the force at flexion of 90° was 39% among the four patients (Table 2). The greatest force was recorded in the third patient (804 N), for whom the degree of leg-lengthening was similar or less than the other three patients. However, the patient’s hip was very tight when extended. The postoperative course was uneventful for all four patients.

**Table 2:** Measured hip joint forces in the four patients

		Maximum force (N)	Minimum force (N)	Flexion angle at the minimum force (°)	Force at 90 flexion (N)
Patient 1	Mean	312.0^*^	116.9	30.0	279.8
	Range	312.0^*^	105.5 – 128.4	28.0—33.0	249.6—283.8
	COV(%)	^*^	7.4	7.2	8.3
Patient 2	Mean	384.7	135.0	39.4	174.9
	Range	348.2—437.8	122.1—143.2	45.0—35.4	164.6—182.3
	COV (%)	12.2	8.4	10.3	5.3
Patient 3	Mean	791.2	134.5	46.2	222.5
	Range	779.5—804.2	132.8—137.0	43.8—50.4	210.4—233.9
	COV (%)	1.6	1.6	6.2	5.3
Patient 4	Mean	186.9	93.0	15.7	155.1
	Range	182.9—194.5	84.8—97.2	13.6—17.1	152.8—158.2
	COV (%)	3.5	7.6	9.5	1.8
Mean		418.7	120.0	32.8	204.5
Mean intrasubject COV (%)		5.8	6.5	8.3	4.7
Intersubject COV (%)		58.3	16.5	40.2	24.2

^*^Data was limited or not available due to errors. COV% = The coefficient of variation

### Musculoskeletal model simulation

**Fig. 7:**
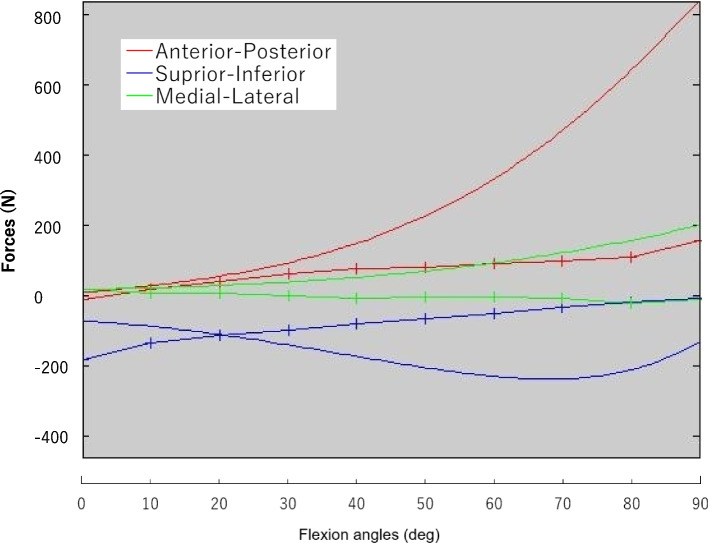
The hip joint forces were calculated by the musculoskeletal model (simple solid lines) and the mean of the measured values of patient 4 (lines with crosses)

The hip joint forces calculated by the musculoskeletal model of the fourth patient are shown in Fig. 7. During hip extension-flexion, general patterns of calculated hip joint forces were roughly consistent with those of the intraoperative measurements. However, some discrepancies were observed. For example, in extension, the measured superior directed forces were larger than the calculated forces, while in flexion the measured posteriorly directed forces were smaller than the calculated forces.

## Discussion

In the present study, intraoperative soft-tissue tension and force direction around the hip were objectively measured in four patients using a novel sensor-instrumented modular femoral head. In addition, the measured forces were compared to theoretical calculations using a musculoskeletal computer model.

Although there are several reports about device ideas for instrumented prostheses for the hip [17, 20] and postoperative joint force measurements [2, 21], this is the first report of consecutive intraoperative soft tissue tension measurements during extension-flexion movements. The lack of previous reports about such measurements, motivated us to provide context for the measured forces using a musculoskeletal computer model.

Within-individual variations of soft-tissue tension, direction, and pattern of force were small during three repeated cycles of measurement (mean COV% was less than 7%), and the within-individual variations in force direction were also small (mean COV% was less than 2%). These observations suggest that a force sensor can be used reliably during surgery. However, soft-tissue tension magnitude varied significantly between subjects (mean inter-subject COV% was 16 to 58%), especially for the maximum resultant hip force in the extended hip. The largest force was recorded in the third patient (804 N), and the hip was tight when extended. If the third patient was excluded, the mean inter-subject COV% was 36%, which is still a wide variation in soft-tissue tension between patients.

In order to theoretically calculate the hip joint forces using a musculoskeletal model, two issues are important. First, suitable measurements are required. In the present study, intraoperative soft-tissue tension during extension-flexion movements was obtained. Successful intraoperative measurements demonstrated that the force sensor could work in vivo and is feasible for surgical applications in a sterile field. In the previous study, our sensor-instrumented modular femoral head was calibrated on a material testing machine (Instron 4204, Instron, Norwood, MA, USA). In that calibration, the force sensor was loaded from three-dimensional directions (30° < θ < 90°, 0° < ϕ < 45°, 15° increments) three times. The magnitude and the angle of the applied loads bounded those measured by the instrumented head. The calibration study showed that output signals were linearly related to the applied force when the force was applied to the sensor. This benchtop calibration showed mean absolute errors of force magnitude were 2.87%, and mean absolute angular errors were 1.44° [28]. Second, patient-specific models should be used to approximate the loading conditions [10]. Although the lower body model used in the present study was scaled to one patient’s anatomy, and the hip alignment was also adjusted to the prosthesis configuration, the soft-tissue conditions such as muscle stiffness could not be adjusted objectively. We observed discrepancies between the measured forces and the calculated forces that might be attributed to characteristics of patients with hip diseases and leg-length anomalies that are different from the healthy adults on which the OpenSim model was developed [5, 6]. We have previously reported using a different musculoskeletal model [12] that used subject-specific three-dimensional CAD models and produced closer agreement for computed joint forces and for force sensing prosthesis. We chose to use the OpenSim framework for this study because it provides a community-standard and convenient method.

The limitations of the study are as follows: First, our study quantified hip joint forces prior to capsular and external rotator repair, which are performed to reduce the dislocation rate when using the posterolateral approach [31]. It was necessary for us to measure soft-tissue tension before capsular and external rotator repair because the final metal femoral head was attached to the neck of the femoral stem after the measurements. Second, the musculoskeletal model calculation was run for patient 4 only. Although individual modeling was possible based on the bone and implant geometries, there was no information on soft tissue. Therefore, the joint forces were almost identical for all patients. Patient 4 was selected because it had the closest body information to the average (Table 1). Third, our final goal in developing the sensor-instrumented modular femoral head was to develop a clinically useful method to quantify soft-tissue tension during THA surgery. To be of practical use, the sensing device needs to be easy to apply in surgery. Our prototype sensor has electric wires for data communication and a power supply between the sensor and a data logger, which was placed outside the surgical area. Thus, the system requires further development to implement wireless communication and real-time display before it can be practically applied. In the present study, the hip joint angle was based on recorded videos, although computer navigation systems permit more accurate hip joint angle measurements.

## Conclusions

With this sensor-instrumented modular femoral head, soft-tissue tension and force direction can be measured objectively during THA surgery. In addition, the measured forces were compared using a musculoskeletal computer model. Soft-tissue tension and force direction are significantly changed from hip extension to hip flexion. With the hip flexed to 90°, soft-tissue tension produced compression force in the antero-posterior direction, and this is related to the incidence of posterior dislocation. We believe that measurements of subjective soft-tissue tension will be useful for enhanced understanding of dislocation mechanisms, to permit optimized intraoperative soft-tissue balance, and ultimately to decrease the incidence of dislocation after THA.
