# Correction: Soft‑tissue tension during total hip arthroplasty measured in four patients and predicted using a musculoskeletal model

**DOI:** 10.1186/s40634-023-00712-x

**Published:** 2023-12-28

**Authors:** Masaru Higa, Hiromasa Tanino, Hiroshi Ito, Scott A. Banks

**Affiliations:** 1 Department of Mechanical Engineering, University of Hyogo, Shosha 2167, Himeji, Hyogo 671‑2280 Japan; 2 Department of Orthopaedic Surgery and Arthroplasty, Asahikawa Medical University, Asahikawa, Hokkaido Japan; 3 Department of Mechanical & Aerospace Engineering, University of Florida, Gainesville, FL USA

**Correction: J Exp Orthop 10, 130 (2023)**

10.1186/s40634-023-00689-7.

Following publication of the original article [1], the author reported that the authors’ affiliation assignment was incorrect.

Masaru Higa^1,2,3^, Hiromasa Tanino^1,2,3^, Hiroshi Ito^1,2,3^ and Scott A. Banks^1,2,3^

The correct version is shown in the authorgroup section above and the original article has been corrected.
